# Survey of interdigital phlegmon outbreaks and their risk factors in free stall dairy herds in Finland

**DOI:** 10.1186/s13028-017-0313-0

**Published:** 2017-07-12

**Authors:** Miia Kontturi, Minna Kujala, Reijo Junni, Erja Malinen, Eija Seuna, Sinikka Pelkonen, Timo Soveri, Heli Simojoki

**Affiliations:** 1 Department of Production Animal Medicine, Faculty of Veterinary Medicine, University of Helsinki, Paroninkuja 20, 04920 Saarentaus, Finland; 2 Veterinary Bacteriology Research Unit, Finnish Food Safety Authority Evira, Mustialankatu 3, 00790 Helsinki, Finland; 3 Veterinary Bacteriology Research Unit, Finnish Food Safety Authority Evira, Neulaniementie 4, 70210 Kuopio, Finland

**Keywords:** Interdigital phlegmon, Outbreak, Risk factors, Infectious hoof diseases, Foot rot, Foul in the foot, Interdigital necrobacillosis

## Abstract

**Background:**

Severe outbreaks of interdigital phlegmon (IP) associated with a high morbidity and major economic losses have occurred in Finland in the past decade. A survey was performed to indicate the current occurrence of infectious hoof diseases and to identify herd level risk factors predisposing to an outbreak of IP.

**Results:**

Responses to a questionnaire revealed that an outbreak of IP defined as morbidity ≥5% within the 1st month of the outbreak, had occurred in 18.0% of the respondent study farms. Risk factors for an outbreak included animal transport between herds, i.e. either animal purchase or contract heifer rearing, enlargement or renovation of the barn, and if the fields of the farm had been organically cultivated. Having any kind of mechanical ventilation in comparison to natural ventilation seemed to lower the risk of IP. Additionally, the farms that had experienced an outbreak of IP often had other infectious hoof diseases. However, it was unclear which disease appeared first.

**Conclusions:**

More attention is needed before and during enlargement or renovation of the barn and substantial planning is crucial for every part of the enlargement process in dairy farms.

## Background

Recently, several dairy herds in Finland experienced a sudden outbreak of interdigital phlegmon (IP). These outbreaks mainly occurred in recently built or renewed free stall barns and caused major economic losses due to high morbidity, antibiotic treatment of the affected cows and discarded milk [1]. No preceding trauma to the interdigital cleft of the affected cows has been reported. Previously, only a few cases of infectious hoof diseases were detected in Finnish dairy farms.

At the same time, the structure of dairy industry in Finland has changed; based on the statistics of National Resources Institute Finland the average number of lactating cows per herd has more than doubled during last 15 years, and the change from tie stalls to free stalls have also occurred simultaneously. In addition, several new techniques have been introduced in the farms, like mixed ration feeding and automatic milking system.

A lot of research has been done on the aetiology and possible risk factors of IP. *Fusobacterium necrophorum* is considered a major pathogen in IP [2, 3] although several other bacteria and environmental factors influence the development of disease [4, 5]. Typically, the first sign of IP is slight lameness, which becomes more apparent when the infection progresses. A swelling of the interdigital area and the bulbs of the heels together with a fetid odour are regarded as characteristic. Soon a fissure forms with swollen protruding edges along the interdigital cleft. In severe cases, systemic signs may appear, which include fever, recumbency, anorexia and decrease in milk production [6]. IP reduces milk yield [7] and can result in an early culling of the affected cow [7, 8].

In general, IP occurs as a sporadic disease in cattle. The frequency or prevalence of IP has been reported being 0.2–5% in herds in North America [9–11], but some other reports of earlier outbreaks of IP exist where the incidence may be as high as 17–25% [12, 13].

As long ago as 1945, Johnson [14] reported that the increase in the incidence of IP is associated with increased animal traffic between herds and cases occur when cattle are introduced to a pasture with alfa–alfa hay or clover. A trauma to the interdigital skin and muddy conditions were mentioned as predisposing factors for the disease. Other studies have also investigated a preceding trauma and muddy or moist conditions underneath the hooves as predisposing factors [4, 6]. In addition, Gupta et al. [6] mentioned seasonality, intensive farm practice, concrete flooring and coarse sand. A Danish study reported cow-level risk factors that included calving, first parity and free stall housing in comparison to tie stall housing [15].

However, most of this research on IP is quite old or does not focus on the actual outbreaks. In response to numerous outbreaks of IP in dairy herds in Finland, we wanted to investigate the possible herd level risk factors and furthermore, to describe the current situation of infectious hoof diseases in Finland.

## Methods

### Study population

In the spring of 2013, a questionnaire regarding infectious hoof diseases was sent to all Finnish dairy farms in a dairy herd recording database that had ≥50 cows in 2012 (n = 1134). The total number of comparable dairy herds in Finland in the spring of 2013 was 1245 [16] and therefore, our initial target population covered 91.1% of the herds of similar size in Finland. The contact data was received from ProAgria agricultural data processing center. Only free stall herds were included in the study.

### Study questionnaire

The final draft of the five-page questionnaire contained 35 questions of general herd data, which included herd size, mean milk yield and region, and questions about the barn characteristics and herd management details. The key items asked are listed in Table 1. The number of the cow compartments meant the number of barns or separate sections of a barn for milking cows. Some farmers separate their milking cows for example based on udder health or milk yield. The flooring choices in the questionnaire were slatted or solid with or without rubber, where the choice ‘with rubber’ included rubber everywhere or only partly in the alleys or manure pack flooring in non-insulated free stall barns. The ventilation system choices contained natural or mechanical systems with or without an additional precision ventilation of the manure drain.

**Table 1:** Summary of the herd and barn characteristics and management practices included in the questionnaire

Herd and barn characteristics	Management practices
Herd size	Mixed ration feeding
Mean milk yield	Outdoor access during summer
Region	Outdoor access during winter
Number of cow compartments	Hoof trimming frequency
Stall type (freestall or tiestall)	Stocking density
Free stall type (insulated, partly insulated, non-insulated)	Enlargement or renovation of the barn
Flooring (slatted or concrete and with or without rubber)	Animal purchase
Milking in a parlour or an automatic milking system	Contract heifer rearing
Ventilation system	Fields in organic farming

The mixed ration feeding included total or partial mixed ration. One question covered any possible enlargement or renovation of the barn in 3-year period prior to the outbreak of IP or 3 year period prior to answering the questionnaire. Two questions inquired about the animal transport between herds i.e. the animal purchase during the past year and information about where the heifers of the herd are kept. Some farms in Finland do not raise their own heifers and instead their calves are sent to another herd, where they are kept until the animals return to the home herd before the first calving. In general, Finnish contract heifer rearing units have animals from several independent farms. Another question inquired about whether the fields of the farm were cultivated organically.

Additionally, the dairy farmers were asked several questions about the leg and claw health in their herd. First, they had to clarify whether they had experienced (1) an outbreak of IP in the last 10 years, in other words had several cases of IP in a short period, or (2) had only a few cases of IP, or (3) had no cases of IP at all. The common signs of IP were described as a list. They included fever, lameness, swelling above a hoof, shaking of a leg, bad odour in the hoof region, and lesions in between the hooves or in the heel. The farmers had to choose the signs exhibited by their affected animals and record the number of the affected cows within first 2 weeks of the outbreak.

Second, we inquired about the signs of interdigital dermatitis (ID), digital dermatitis (DD), interdigital hyperplasia (IH) and verrucous dermatitis. Similarly, the signs of ID and DD were listed and included lesions between the hooves or in the heel and reddish, painful lesions in the hoof region. Third, we asked for the dairy farmer’s observations about whether the herd had occurrences in excess of 5% white line lesions that needed a hoof blocking for treatment and any visual signs of calf diphtheria i.e. buccal abscesses in suckling calves.

Most of the cases of IP in Finland are diagnosed and treated by veterinarians, because of a strict national antibiotic policy. In the outbreaks, the correct diagnosis is achieved relatively straightforward. Therefore, we expected the IP status given in the questionnaire to be accurate. On the other hand, ID and DD are rarer and their signs are confusing, so we predicted more variation with these answers. Because of this unpredictability and possible confusion of the farm staff in making the correct diagnosis of ID and DD we considered the existence of any of their listed signs as other infectious hoof disease than IP.

The questionnaire was pilot tested with two dairy veterinarians and producers and modified based on their feedback. The final drafts of the questionnaire were mailed to dairy farmers with an enclosed paid return envelope. An alternative option was to answer the same questionnaire online. Furthermore, all dairy veterinarians in Finland were informed about the survey and asked to remind their dairy farmer clients. No separate reminders were sent to farmers themselves. After the return of the questionnaires, some of the farms were called to clarify certain answers.

### Statistical analyses

We collated and inserted the data from the original paper document questionnaire into Excel spreadsheets and used Stata IC version 14 (Stata Corporation, Texas, USA) for statistical analyses. Only free stall barns with a complete information on the IP status of the herd were included in the analyses. A *P* value <0.05 was considered statistically significant. The descriptive data are presented as percentages. The possible outbreak of IP associated putative disorders were tested using the Chi squared test.

Our study herds were categorized into three groups according to the IP status of the herd, thus: (1) outbreak of IP, (2) few cases of IP, and (3) no IP in the herd. The herds with no IP are hereafter referred to as the control herds. An outbreak of IP was defined as morbidity due to IP being ≥5% during the 1st month of the outbreak.

The predictors included herd size, milk yield, region and all variables listed in Table 2. Herd size was a continuous variable. Data on herd size were not normally distributed, thus they were divided into three herd size classes: (1) 50–65, (2) 66–99, and (3) ≥100 cows per herd. Milk yield was a normally distributed continuous variable and was presented as 1000 kg in analyses. The geographical location of the herd was categorized into four groups based on counties in Finland; (1) southern, (2) western, (3) eastern, and (4) northern region. The flooring variable was categorized into (1) slatted concrete, (2) solid concrete, (3) slatted floor with rubber, and (4) solid floor with rubber. The ventilation system was divided in two groups; (0) natural ventilation, (1) natural ventilation with an additional precision ventilation of the manure drain or mechanical ventilation with or without an additional precision ventilation of the manure drain. This latter option is later referred as a mechanical ventilation system.

**Table 2:** Descriptive statistics of the study herds (n = 355)

Variable	Replies (n)^1^	Control (%)	Few cases of IP (%)	Outbreak of IP (%)	Total (%)
Study herds (n)	*355*	*259* (*73*.*0*%)	*32* (*9.0*%)	*64* (*18.0*%)	*355* (*100*%)
Region	353				
Southern		37 (14.4%)	2 (6.2%)	5 (7.8%)	44 (12.4%)
Western		116 (45.1%)	14 (43.8%)	33 (51.6%)	163 (46.2%)
Eastern		55 (21.4%)	8 (25.0%)	12 (18.7%)	75 (21.3%)
Northern		49 (19.1%)	8 (25.0%)	14 (21.9%)	71 (20.1%)
Barn characteristics					
Number of cow compartments in the barn	355				
1		218 (84.2%)	24 (75.0%)	36 (56.3%)	278 (78.3%)
≥2		41 (15.8%)	8 (25.0%)	28 (43.7%)	77 (21.7%)
Free stall type	345				
Insulated		198 (78.6%)	19 (61.3%)	36 (58.1%)	253 (73.3%)
Partly insulated		42 (16.7%)	11 (35.5%)	23 (37.1%)	76 (22.0%)
Non-insulated		12 (4.7%)	1 (3.2%)	3 (4.8%)	16 (4.7%)
Flooring	341				
Slatted concrete		120 (48.2%)	13 (41.9%)	28 (45.9%)	161 (47.2%)
Solid concrete		62 (24.9%)	10 (32.3%)	17 (27.9%)	89 (26.1%)
Slatted rubber		17 (6.8%)	1 (3.2%)	4 (6.5%)	22 (6.5%)
Solid rubber		50 (20.1%)	7 (22.6%)	12 (19.7%)	69 (20.2%)
Milking system	345				
Milking parlour		121 (48.0%)	11 (35.5%)	22 (35.5%)	154 (44.6%)
Automatic milking system		131 (52.0%)	20 (64.5%)	40 (64.5%)	191 (55.4%)
Ventilation system	343				
Natural		56 (22.2%)	10 (32.3%)	30 (50.0%)	96 (28.0%)
Mechanical^2^		196 (77.8%)	21 (67.7%)	30 (50.0%)	247 (72.0%)
Management practices					
Total or partial mixed ration feeding	355				
No		140 (54.1%)	7 (21.9%)	13 (20.3%)	160 (45.1%)
Yes		119 (45.9%)	25 (78.1%)	51 (79.7%)	195 (54.9%)
Outdoor access during summer	355				
No		154 (59.5%)	17 (53.1%)	47 (73.4%)	218 (61.4%)
Yes		105 (40.5%)	15 (46.9%)	17 (26.6%)	137 (38.6%)
Outdoor access during winter	353				
No		214 (82.6%)	23 (76.7%)	55 (85.9%)	292 (82.7%)
Yes		45 (17.4%)	7 (23.3%)	9 (14.1%)	61 (17.3%)
Hoof trimming frequency	355				
0–1		80 (30.9%)	8 (25.0%)	20 (31.2%)	108 (30.4%)
≥2		179 (69.1%)	24 (75.0%)	44 (68.8%)	247 (69.6%)
Stocking density (cows/stall)	345				
<1		90 (35.9%)	10 (32.2%)	34 (54.0%)	134 (38.8%)
1		132 (52.6%)	14 (45.2%)	23 (36.5%)	169 (49.0%)
>1		29 (11.5%)	7 (22.6%)	6 (9.5%)	42 (12.2%)
Enlargement^3^ within 3 years	349				
No		180 (69.8%)	14 (50.0%)	21 (33.3%)	215 (61.6%)
Yes		78 (30.2%)	14 (50.0%)	42 (66.7%)	134 (38.4%)
Open or closed herd	353				
Closed herd		189 (73.3%)	17 (53.1%)	24 (38.1%)	230 (65.2%)
Open herd		69 (26.7%)	15 (46.9%)	39 (61.9%)	123 (34.8%)
Fields in organic farming	355				
No		242 (93.4%)	27 (84.4%)	55 (85.9%)	324 (91.3%)
Yes		17 (6.6%)	5 (15.6%)	9 (14.1%)	31 (8.7%)

*IP* interdigital phlegmon

^1^The column ‘Replies (n)’ describes the number of replies given to each question

^2^Natural with additional precision ventilation of the manure drain or mechanical with or without additional precision ventilation of the manure drain

^3^Enlargement or renovation of the barn

The enlargement or renovation of the barn was a dichotomous variable, where (0) no enlargement or renovation in 3 years prior to the outbreak in outbreak herds or no enlargement or renovation in 3 years’ prior replying to the survey in control herds and (1) enlargement or renovation in 3 year period prior to the outbreak in outbreak herds or enlargement or renovation during the 3 year period prior to replying to the survey in control herds. The open or closed herd variable discriminated between two groups; (0) closed herd i.e. no purchase of cattle and no contract heifer rearing, (1) open herd i.e. purchase of cattle or contract heifer rearing or both.

The sample size in the model was 294 herds with an occurrence of an outbreak of IP 19%. An odds ratio (OR) 2.7 with a power of 0.9 for predictors was determined as significant for this sample size, assuming the proportion of exposed controls for this predictor was 25%.

### Statistical model

We excluded the herds with few cases of IP from the statistical analysis of the risk factors of an IP outbreak. Associations between all predictors and the outcome were computed using simple logistic regression. The predictors with association ≤0.2 were included in the full model. A manual stepwise backwards procedure was used to build the nested model. Removed variables were evaluated at each step for confounding effects by checking if the coefficients for remaining variables had changed over 20%. The region, herd size and the milk yield were considered to be confounding variables and therefore, were kept in the model. Moreover, full and nested models were compared with the logistic likelihood ratio test.

We tested all biologically plausible interactions, but detected no significant association. We also evaluated the model by sensitivity and specificity test and roc-curve of the model. The assumptions of the model were controlled by normality and scatter plots of the model residuals. One herd was found to be an outlier, but because it did not change the results essentially, it was kept in the model.

## Results

A total of 390 questionnaires were returned resulting in a response rate of 34.4%. Of these responses, 355 free stall herds had complete information on their IP status and therefore were included in the study.

The mean herd size was 79.8 dairy cows (range 50–293.7) and the mean yearly milk yield of the herds was 9364 kg (6800–12,500 kg). Table 2 summarizes the barn characteristics and management practices of the study herds.

**Fig. 1:**
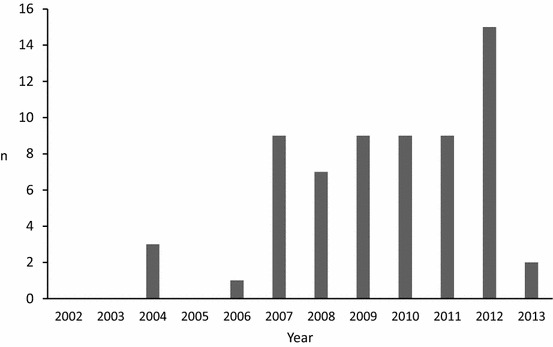
Number of the herds with an outbreak of interdigital phlegmon (IP) per year. The number (n = 64) of the herds with an outbreak of IP based on the replies to our survey. The survey was performed in May 2013 and therefore, the last year in this chart is incomplete

Sixty-four out of 355 herds (18.0%) had had an outbreak of IP with morbidity ≥5% within the 1st month of the outbreak. All reported outbreaks had occurred in the year 2004 or later and a majority (68.8%) within the 2009–2013 period (Fig. 1). Furthermore, 32 (9.0%) respondents reported few cases of IP in the herd. Out of 64 IP herds 14 (21.9%) reported cases of IP in heifers and 11 (17.2%) in calves. A year after the outbreak, in 21 out of 47 outbreak herds (44.7%) still had sporadic cases of IP in cows, in 4 herds (8.5%) in heifers, and in 1 herd (2.1%) in calves. Signs of IP cases in the 64 outbreak herds were as follows: 33 (51.6%) herds reported fever, 62 (96.9%) lameness, 62 (96.9%) swelling above a hoof, 36 (56.3%) shaking of a leg, 34 (53.1%) bad odour in the hoof region, 50 (78.1%) lesions in between the hooves and 25 (39.1%) lesions in the heel.

### Risk factors for an outbreak of IP

We compared control farms (n = 239) and farms with an outbreak of IP (n = 55) and analyzed the associations of various predictors. Out of 323 herds, we included 294 herds with a complete dataset in the analysis. Table 3 presents the results of a logistic regression model.

**Table 3:** Final logistic regression model for an outbreak of interdigital phlegmon (IP)

Variable	n	Odds ratio	*P* value	95% CI^1^	Wald^2^
Free stall type					
Insulated	221	Referent			0.09
Partly insulated	60	0.31	0.06	0.09–1.05	
Non-insulated	13	0.14	0.07	0.02–1.20	
Ventilation					
Natural	84	Referent			
Mechanical^3^	210	0.18	<0.01	0.06–0.54	
Herd size					
50–65	125	Referent			0.07
66–99	115	0.97	0.94	0.41–2.26	
≥100	54	2.60	0.04	1.03–6.58	
Enlargement^4^ within 3 years					
No	184	Referent			
Yes	110	3.32	<0.01	1.58–6.98	
Open or closed herd					
Closed herd	194	Referent			
Open herd	100	4.91	<0.01	2.32–10.37	
Fields in organic farming					
No	270	Referent			
Yes	24	3.75	0.02	1.21–11.66	
Milk yield^5^	294	0.73	0.18	0.45–1.16	
Region					
Southern	38	Referent			0.56
Western	138	1.75	0.40	0.48–6.37	
Eastern	62	2.10	0.30	0.51–8.65	
Northern	56	2.77	0.17	0.65–11.82	
_Constant		1.88	0.80	0.02–218.92	

The associations of various herd level variables and an outbreak of IP. The number of the outbreak herds in the model is 55 and control herds 239 (n = 294)

^1^95% CI = 95% confidence interval

^2^Wald-test was used to test the overall *P* value of the variable

^3^Natural with additional precision ventilation of the manure drain or mechanical with or without additional precision ventilation of the manure drain

^4^Enlargement or renovation of the barn

^5^Milk yield is in 1000 kg

### Occurrence of an outbreak of IP associated putative diseases

**Fig. 2:**
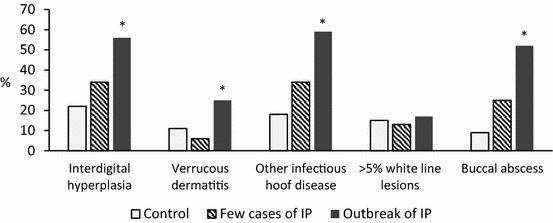
Interdigital phlegmon (IP) associated putative diseases and occurrence (%) in various disease groups. The occurrence of various diseases in group with no outbreak of IP i.e. control herds (n = 259), group with few cases of IP (n = 32) and group with an outbreak of IP (the morbidity of IP ≥5% during the 1st month of the outbreak, n = 64). The definition of the other infectious hoof diseases includes lesions in between the hooves or in the heel and reddish, painful lesions in the hoof region. The >5% white line lesions describes lesions that needed a hoof blocking for treatment, and buccal abscesses characterize visual signs of calf diphtheria in suckling calves. The Chi squared test was used between control and outbreak herds and significant difference is marked with *P < 0.01

We studied the association of an outbreak of IP with the occurrence of various other hoof diseases and visual signs of calf diphtheria such as buccal abscesses. Interdigital hyperplasia, verrucous dermatitis and other infectious hoof diseases, like ID and DD, were detected more frequently (Pearson’s Chi^2^ 28.6, 10.4, and 46.4 respectively, in all *P* < 0.01) among the herds with an outbreak of IP (Fig. 2). Similarly, buccal abscesses in suckling calves were observed more often (Pearson’s Chi^2^ 65.3, *P* < 0.01).

## Discussion

We determined the current situation regarding infectious hoof diseases in Finland. Eighteen percent of the herds who responded to the questionnaire in our study were diagnosed as having an outbreak of IP with morbidity ≥5% within the 1st month of the outbreak, whereas 9.0% of the herds who reported having sporadic cases of IP did not fulfill our definition of an outbreak. Interestingly, those farms that had experienced an outbreak of IP, often had other infectious hoof diseases. However, it was impossible to deduce from our study design and survey data, which disease appeared first in the herd. Likewise, a study from the Netherlands reported that the presence of other infectious hoof disorders, such as interdigital dermatitis and heel horn erosion (IDHE) and IP, increased the risk for DD, and IP appeared to be strongly associated with DD (OR = 4.4) [17]. Other reports of correlations between other infectious hoof diseases than IP also exist [18–20]. Recently, veterinary practitioners in Finland have observed an increase in the number of buccal abscesses in young dairy calves in herds with a previous outbreak of IP. Our survey data supports this observation. A buccal abscess may characterize a sign of calf diphtheria; a respiratory tract disease where *F. necrophorum* plays a part in the pathogenesis [21, 22].

We investigated the possible herd level risk factors for an outbreak of IP to occur. Our study determined recent enlargement or renovation of the barn to be a risk factor for an outbreak of IP. During the enlargement process, both the cattle and the dairy producers suffer from stress; they must adapt to a new building, and management regime that differ from the old system. Before the introduction of the new free stall, some overstocking may occur in the old building, which would plausibly increase the infection pressure. Occasionally, when the cattle are compelled to move into new free stall, the facility can still be a construction site, which might increase the risk for injuries and is an additional burden and stress factor to farmers. As part of the restructuring and the enlargement, the tradition of the family managed farm may end, and the cattle in the enlarged herds will be managed by employed staff. If the management is not of sufficient quality or there are fewer stockmen to look after more cows, the risk of mistakes and unnoticed signs of ill-health may increase.

We also determined the animal transport between herds to be a risk factor for an outbreak of IP. This was not surprising, because of the infectious nature of the disease [3, 6]. Moreover, the purchase of new animals occurs more frequently during the enlargement of a herd, and contract heifer rearing is also more common before or after the enlargement when producers try to adapt to different management strategies to deal with the increasing herd size. Buying in new heifers from other farms has been reported as a risk for DD [23–25]. On the other hand, a new animal in a herd always meets different environment and management, which may affect its immunology. The possible causality of the animal purchase and the IP outbreak in our study was unclear in the questionnaire data and therefore, purchase of heifers may not be a causal factor in the IP outbreak but simply be a simultaneous management course of action in the farm.

We also found that organically cultivated fields were a risk factor. Even though the number of the herds with organically cultivated fields (n = 24) was small in our model, the post hoc power analysis (0.88) showed that the group was big enough to detect the possible risk. In Finland, the soil lacks selenium (Se), and in conventional farming the use of fertilizers with Se supplement is standard. However, the regulations of organic farming limit the use of fertilizers and if Se supplement is not added in the feed, a deficiency might occur. Low blood Se level might alter the immunological status of the cows and increase disease susceptibility. For example, some studies have detected an association with low Se and vitamin E deficiency and udder health [26, 27]. Another possible underlying cause in organic farming may be the excessive use of clover in the silage. Johnson [14] speculated as long ago as 1945 that clover might be a reason for an increased incidence of IP. Moreover, due to the EU regulations of organic farming (Council regulation 2007/834/EC) animal purchase is more likely to happen between other organic herds. The risk of IP spreading from one organic farm to another is increased if the IP is more common in organic farms than conventional ones. None of these possible factors listed above could be ascertained by only one question in this survey. More details about the grazing conditions, trace element status and grass and clover species content of fields would be needed in future studies to investigate such possible factors.

Any form of mechanical ventilation was found to decrease the risk of an outbreak of IP. We presume that the better the ventilation the drier the indoor air and less ammonia in the free stall. Furthermore, the drier the indoor air the cleaner the cows are. Two Norwegian studies reported that barns with low air humidity had cleaner animals [28] and for each 10% increase in relative air humidity, the risk of dirty thighs increased [29]. In the past, moist conditions underneath the hooves have been associated with IP [4, 6]. Several studies have also found associations between other infectious hoof diseases and dirty claws or legs or dirty alleys [20, 25, 30, 31]. The flooring type had no effect on the outbreaks of IP in our study in contrast to studies of DD from the UK and USA. Solid flooring of grooved concrete was identified as a risk factor for DD in the UK [32] and in the USA [24] when compared to textured concrete floors.

In this study, an overstocking was not a risk for an outbreak of IP. However, the animal protection law in Finland prohibits an overstocking and one stall per cow is compulsory. This may have affected the responses of farmers. Additionally, the stocking situation in the barn varies constantly.

Replies to our survey represented the regional differences in dairy farming in Finland quite well; all areas were represented and replies from areas with more dairy cows, like provinces Pohjanmaa and Savo, were a greater number. Additionally, the mean milk yield of the study herds related appropriately to free stall herds of similar size. However, some bias may exist in our results. Presumably the farms that had problems with infectious hoof diseases were more prone to answer the survey and therefore, our results may be moderately skewed. Although we could detect several possible risk factors for an outbreak of IP, we were not able to specify why there has been so many of these outbreaks in Finland. One reason could be that other countries had experienced similar outbreaks much earlier [12, 13, 33], and due to Finland’s relative isolation Finnish dairy herds are still immunologically quite naïve and therefore more susceptible to IP.

## Conclusions

This study revealed risk factors that were associated with the outbreaks of IP in Finland. These risk factors were animal transport between herds, enlargement or renovation of the barn and organic cultivation of the fields. However, having a forced ventilation system in the free stall barn lowered the risk. These results suggest that more attention is needed before and during the enlargement process and substantial planning is crucial in every part of that undertaking. Since the farms that had experienced an outbreak of IP, often had other infectious hoof diseases as well we speculated that this strategic approach would additionally reduce the incidence of these further diseases.

## References

[1] Häggman J, Junni R, Simojoki H, Juga J, Soveri T (2015). The costs of interdigital phlegmon in four loose-housed Finnish dairy herds. Acta Vet Scand.

[2] Flint JC, Jensen R (1951). Pathology of necrobacillosis of the bovine foot. Am J Vet Res.

[3] Clark BL, Stewart DJ, Emery DL (1985). The role of *Fusobacterium necrophorum* and *Bacteroides melaninogenicus* in the aetiology of interdigital necrobacillosis in cattle. Aust Vet J.

[4] Johnson DW, Dommert AR, Kiger DG (1969). Clinical investigations of infectious foot rot of cattle. J Am Vet Med Assoc.

[5] Berg JN, Loan RW (1975). *Fusobacterium necrophorum* and *Bacteroides melaninogenicus* as etiologic agents of foot rot in cattle. Am J Vet Res.

[6] Gupta RB, Fincher MG, Bruner DW (1964). A study of the etiology of foot-rot in cattle. Cornell Vet.

[7] Hernandez J, Shearer JK, Webb DW (2002). Effect of lameness on milk yield in dairy cows. J Am Vet Med Assoc.

[8] Booth CJ, Warnick LD, Gröhn YT, Maizon DO, Guard CL, Janssen D (2004). Effect of lameness on culling in dairy cows. J Dairy Sci.

[9] DeFrain JM, Socha MT, Tomlinson DJ (2013). Analysis of foot health records from 17 confinement dairies. J Dairy Sci.

[10] Cramer G, Lissemore KD, Guard CL, Leslie KE, Kelton DF (2008). Herd- and cow-level prevalence of foot lesions in Ontario dairy cattle. J Dairy Sci.

[11] Oberbauer AM, Berry SL, Belanger JM, McGoldrick RM, Pinos-Rodriquez JM, Famula TR (2013). Determining the heritable component of dairy cattle foot lesions. J Dairy Sci.

[12] David GP (1993). Severe foul-in-the-foot in dairy cattle. Vet Rec.

[13] Doherty M, Bassett H, Markey B, Healy A, Sammin D (1998). Severe foot lameness in cattle associated with invasive spirochaetes. Ir Vet J.

[14] Johnson KL (1945). Infectious pododermatitis in dairy cattle. N Am Vet.

[15] Alban L, Lawson LG, Agger JF (1995). Foul in the foot (interdigital necrobacillosis) in Danish dairy cows—frequency and possible risk factors. Prev Vet Med.

[16] National Resources Institute Finland: number of dairy cows in various herd sizes 1.5.2012. http://stat.luke.fi/en/number-bovine-animals-1-may-2012_en. Accessed 20 Apr 2017.

[17] Holzhauer M, Hardenberg C, Bartels C, Frankena K (2006). Herd- and cow-level prevalence of digital dermatitis in the Netherlands and associated risk factors. J Dairy Sci.

[18] Manske T, Hultgren J, Bergsten C (2002). Prevalence and interrelationships of hoof lesions and lameness in Swedish dairy cows. Prev Vet Med.

[19] Capion N, Thamsborg SM, Enevoldsen C (2009). Prevalence and severity of foot lesions in Danish Holstein heifers through first lactation. Vet J.

[20] Knappe-Poindecker M, Gilhuus M, Jensen TK, Klitgaard K, Larssen RB, Fjeldaas T (2013). Interdigital dermatitis, heel horn erosion, and digital dermatitis in 14 Norwegian dairy herds. J Dairy Sci.

[21] Ryff JF, Lee AM (1946). The etiology of calf diphtheria. Am J Vet Res.

[22] Panciera RJ, Perino LJ, Baldwin CA, Morton RJ, Swanson JE (1989). Observations of calf diphtheria in the commercial feedlot. Agri-practice.

[23] Rodriguez-Lainz A, Melendez-Retamal P, Hird DW, Read DH, Walker RL (1999). Farm- and host-level risk factors for papillomatous digital dermatitis in Chilean dairy cattle. Prev Vet Med.

[24] Wells SJ, Garber LP, Wagner BA (1999). Papillomatous digital dermatitis and associated risk factors in US dairy herds. Prev Vet Med.

[25] Rodríguez-Lainz A, Hird DW, Carpenter TE, Read DH (1996). Case-control study of papillomatous digital dermatitis in Southern California dairy farms. Prev Vet Med.

[26] Smith KL, Harrison JH, Hancock DD, Todhunter DA, Conrad HR (1984). Effect of vitamin E and selenium supplementation on incidence of clinical mastitis and duration of clinical symptoms. J Dairy Sci.

[27] Jukola E, Hakkarainen J, Saloniemi H, Sankari S (1996). Blood Selenium, vitamin E, vitamin A, and β-carotene concentrations and udder health, fertility treatments, and fertility. J Dairy Sci.

[28] Hauge SJ, Kielland C, Ringdal G, Skjerve E, Nafstad O (2012). Factors associated with cattle cleanliness on Norwegian dairy farms. J Dairy Sci.

[29] Ruud LE, Bøe KE, Østerås O (2010). Risk factors for dirty dairy cows in Norwegian freestall systems. J Dairy Sci.

[30] Somers JGCJ, Frankena K, Noordhuizen-Stassen EN, Metz JHM (2003). Prevalence of claw disorders in Dutch dairy cows exposed to several floor systems. J Dairy Sci.

[31] Relun A, Lehebel A, Bruggink M, Bareille N, Guatteo R (2013). Estimation of the relative impact of treatment and herd management practices on prevention of digital dermatitis in French dairy herds. Prev Vet Med.

[32] Barker ZE, Amory JR, Wright JL, Mason SA, Blowey RW, Green LE (2009). Risk factors for increased rates of sole ulcers, white line disease, and digital dermatitis in dairy cattle from twenty-seven farms in England and Wales. J Dairy Sci.

[33] Cook NB, Cutler KL (1995). Treatment and outcome of a severe form of foul-in-the-foot. Vet Rec.

